# Enhancing cord blood stem cell-derived NK cell growth and differentiation through hyperosmosis

**DOI:** 10.1186/s13287-023-03461-x

**Published:** 2023-10-15

**Authors:** Wei Wen, Xiang Chen, Xin-Yi Shen, Hua-Yu Li, Feng Zhang, Feng-Qi Fang, Xiao-Bing Zhang

**Affiliations:** 1 State Key Laboratory of Experimental Hematology, National Clinical Research Center for Blood Diseases, Haihe Laboratory of Cell Ecosystem, Institute of Hematology and Blood Diseases Hospital, Chinese Academy of Medical Sciences and Peking Union Medical College, Tianjin, China; 2 Tianjin Institutes of Health Science, Tianjin, China; 3 Department of Oncology, The First Affiliated Hospital of Dalian Medical University, Dalian, China; 4 College of Computer Science and Technology, China University of Petroleum (East China), Qingdao, China

**Keywords:** Natural killer (NK) cells, Hematopoietic stem and progenitor cells (HSPCs), Differentiation, Hyperosmosis, Immunotherapy

## Abstract

**Background:**

Natural killer (NK) cells hold great promise in treating diverse hematopoietic and solid tumors. Despite their availability from peripheral blood and cord blood, stem cell-derived NK cells offer an 'off-the-shelf' solution. Hematopoietic stem and progenitor cells (HSPCs) derived from cord blood pose no risk to the newborn or mother and are virtually ideal sources for NK cell differentiation.

**Methods:**

We developed a modified protocol to differentiate HSPCs to NK cells under serum-free conditions using defined factors. The HSPC-derived NK (HSC-NK) cells could be expanded in a K562 feeder cell-dependent manner. Furthermore, using lentivirus transduction, chimeric antigen receptor (CAR)-modified HSPCs could be differentiated into NK cells, leading to the establishment of CAR-NK cells.

**Results:**

The efficiency of NK cell differentiation from HSPCs was increased through the simple modulation of osmotic pressure by the addition of sodium chloride or glucose. Furthermore, the hyperosmosis-primed HSC-NK cells exhibited enhanced proliferation capacity and maintained normal functional characteristics, including transcriptome and antitumor efficacy. The optimized protocol yielded approximately 1.8 million NK cells from a single CD34-positive cell within a 28-day cycle, which signifies more than a ten-fold increase in efficiency relative to the conventional methods. This optimized protocol was also suitable for generating CAR-NK cells with high yields compared to standard conditions.

**Conclusions:**

The results of this study establish high osmotic pressure as a simple yet powerful adjustment that significantly enhances the efficiency and functionality of HSC-NK cells, including CAR-NK cells. This optimized protocol could lead to cost-effective, high-yield NK cell therapies, potentially revolutionizing cancer immunotherapy strategies.

**Supplementary Information:**

The online version contains supplementary material available at 10.1186/s13287-023-03461-x.

## Background

Natural killer (NK) cells are cytotoxic innate lymphoid cells that offer significant potential for immunotherapy [1, 2]. They directly annihilate cancerous or virally infected cells that suppress HLA class I molecules and communicate through cytokine and chemokine production [3]. Remarkably, unlike T cells, NK cells from allogeneic sources, including peripheral blood (PB) and umbilical cord blood (CB), can be safely utilized without the necessity for full HLA matching [4]. Clinical trials have confirmed the safety of allogeneic PB- and CB-derived NK cells, with no incidence of graft-versus-host disease (GVHD) [5], establishing NK cells as promising candidates for 'off-the-shelf' cell therapy products [6].

Chimeric antigen receptor-modified T (CAR-T) cell therapy has emerged as an innovative immunotherapeutic strategy for treating cancers, notably refractory acute lymphoblastic leukemia [7, 8]. However, treatment with autologous CAR-T cells has been linked to significant toxic effects, such as cytokine release syndrome and neurotoxicity [9–11]. Consequently, an effective allogeneic product with a superior safety profile could circumvent these limitations. In this regard, NK cells engineered to express a CAR offer an alternative and potentially safer immunotherapeutic approach for cancer treatment [12].

PB-derived NK cells have a limitation in terms of expansion potential and can expand by hundreds or thousands of times, thus limiting their clinical utility. Stem cell-derived NK cells, specifically induced pluripotent stem cell (iPSC)-derived NK cells, could be a solution to this drawback [13, 14]. These cells can be differentiated from a standardized clone, yielding a uniform NK cell population. Despite this potential, the differentiation and expansion process typically surpass five weeks, with potential accumulated mutations over cell culture and iPSC passage posing a challenge [15, 16]. Nonetheless, this risk could be mitigated using a kill switch, such as inducible CASP9, truncated EGFR or CD20 [17, 18].

An alternative is hematopoietic stem and progenitor cells (HSPCs) derived from cord blood. These are sourced earlier in life with fewer mutations [19] and can be freshly isolated or immediately sourced from readily available off-the-shelf frozen products, posing no risk to the newborn or mother. They can be differentiated into a significant number of highly functional NK cells (HSC-NK) ex vivo within a shorter period of just under four weeks. Given the virtually unlimited supply of cord blood, this source could enable the production of large, quality-controlled batches of NK cells suitable for use in multiple patients.

However, even with their potential to serve as an 'off-the-shelf' source for NK cell production, cord blood HSPCs still pose challenges due to their low differentiation efficiency, making it difficult to generate a sufficient number of homogeneous NK cells for patient treatment from a single batch. Our research aims to overcome this limitation by hypothesizing that a high osmotic pressure environment can enhance both the differentiation efficiency into NK cells and their production yield. By adopting an innovative yet straightforward approach of increasing the osmotic pressure in the culture medium with the addition of substances like sodium chloride or glucose, we have amplified the differentiation efficiency of HSPCs into NK cells and their proliferation rate.

Here, we present an efficient method for generating HSC-NK cells that maintain high proliferation ability and standard tumor-killing activity. Remarkably, over 1.8 × 10^6^ functional HSC-NK cells can be obtained from a single CD34^+^ cell in four weeks. Considering at least 1 × 10^6^ CD34^+^ HSPCs in one CB unit, thousands of doses (1 × 10^7^/kg for 50-kg patients) of NK cells can be obtained from one CB unit.

Moreover, our optimized methodology for HSC-NK cell production holds significant promise for CAR-NK therapy applications. By employing lentivirus-based CAR transduction, we successfully achieved efficient differentiation of HSPCs into HSC-NK cells, with no loss of CAR expression. This validates the feasibility of generating CAR-expressing HSC-NK cells using our optimized method. Notably, our innovative strategy offers the potential to substantially boost NK cell yield, paving the way for broader clinical applications, all without imposing significant additional production costs. Furthermore, this breakthrough also opens the door for the potential application of CAR-NK therapy, harnessing the enhanced functionality and targeting capabilities of CAR-NK cells in treating a range of diseases, particularly cancer.

## Methods

### Primary cell culture

Peripheral blood mononuclear cells (PBMCs), procured from the peripheral blood of healthy donors, were segregated using density gradient centrifugation facilitated by Ficoll-Hypaque (1.077 g/mL). To facilitate PB-NK cell expansion, thawed PBMCs were combined with 2 × 10^5^ K562-mbIL21-feeder cells at a proportion of 1 × 10^5^ cells per ml in StemSpan™ SFEM II medium (Stemcell Technologies). The medium was enriched with 1% L-glutamine (Invitrogen), 100 ng/ml hIL-2 (Peprotech), 20 ng/ml hIL-7 (Peprotech), 20 ng/ml hIL-15 (Peprotech), and 50 μg/ml ascorbic acid (Sigma) [20]. Both the cytokines and ascorbic acid were freshly added prior to use.

Cord blood CD34^+^ HSPCs were isolated using the CD34 MicroBead Kit (Miltenyi Biotec) [21]. The enriched HSPC population comprised over 90% CD34^+^ cells. These HSPCs were introduced at 5 × 10^5^ cells per ml into serum-free StemSpan™ SFEM II medium (Stemcell Technologies). The medium was enriched with 1% L-glutamine (Invitrogen), 100 ng/ml hSCF (Peprotech), 100 ng/ml hFlt3-L (Peprotech), 100 ng/ml hTPO (Peprotech), 50 ng/ml hIL-6 (Peprotech), 750 nM SR1 (Sigma), and 50 nM UM171 (Sigma). To maintain optimal cell density, fresh medium was administered every two days before initiating NK cell differentiation, thus ensuring a cell density range of 5 × 10^5^ to 1 × 10^6^ cells per ml.

### Cancer cell culture

We established green fluorescent protein (GFP)-expressing cell lines (K562, ATCC, CCL-243; Raji, ATCC, CCL-86; HepG2, ATCC, HB-8065; MOLM-13, AddexBio, C0003003/60 and SKOV-3, ATCC, HTB-77) through transduction using a lentiviral vector that encodes the EF1-Puro-2A-GFP cassette. Post-transduction, the cells underwent a puromycin selection phase. We achieved successful establishment of cell lines expressing high GFP levels (> 99%) following this process.

We generated K562-mbIL21 cells through the transduction of a lentiviral vector that expresses mbIL21, CD86 (B7-2), and CD137L (4-1BBL) [22]. High-copy K562-mbIL21 cells were selected based on their membrane-bound IL21 protein expression levels. Using fluorescence-activated cell sorting (FACS), we sorted and selected the top 5% of cells with the highest expression levels, kept them at a low passage, and stored them in a cell bank. Prior to their usage in NK cell stimulation, these cells were inactivated by a 3-h treatment with 10 μg/ml mitomycin C (MMC).

The cell lines K562, Raji, and MOLM-13 were cultured in RPMI-1640 medium, HepG2 cells in MEM medium, and SKOV-3 cells in M5A medium. All culture media were supplemented with 10% fetal bovine serum (FBS) (Gibco) and 1% L-glutamine (Invitrogen).

We transduced mouse OP9 stromal cells (a kind of gift from Tao Cheng’s lab) with lentivirus to express the Notch ligands DLL1 and DLL4 [23]. The establishment of DLL1-2A-Puro-expressing OP9 cells was achieved through lentiviral transduction and subsequent puromycin selection. We then introduced a lentivirus carrying DLL4 and selected the top 5% of cells with the highest expression levels based on DLL4 antibody staining [24]. These transduced OP9 cells were cultivated in MEM supplemented with 20% FBS (Gibco) and 1% L-glutamine (Invitrogen). Prior to utilization, the cells were treated with 10 μg/ml MMC for 3 h in preparation for subsequent experiments.

### Differentiation of hematopoietic stem cells

After the four-day expansion phase of HSPCs, the cells were reseeded at a density of 15,000 cells/ml onto OP9 feeder cells for differentiation [25]. The medium used for differentiation was StemSpan™ SFEM II (Stemcell Technologies), enhanced with 1% L-glutamine (Invitrogen), 20 ng/ml each of hSCF, hFlt3-L, hTPO, hIL-7 (all sourced from Peprotech), and 50 μg/ml ascorbic acid (Sigma) [26]. Cytokines and ascorbic acid were freshly supplemented prior to usage. Generally, the central wells of a TC-24-well plate were employed for differentiation, while the corner and side wells were filled with water. Half of the medium volume was refreshed every three days. After 14 days of expansion and differentiation, both suspension and adherent cells treated with Accutase from Innovative Cell Technologies were combined and analyzed.

To manipulate osmotic pressure, we used either a 9% (w/v) NaCl solution or 10 × PBS. For each 30 mM increment in osmotic pressure, we added 22.4 μl of a 1.5 M NaCl solution to 1 ml of medium. To decrease osmotic pressure, such as to 270 mM, we added 110 μl of water to 1 ml of medium. For non-Na + salt solutions such as KCl, we added 22.4 μl of a 1.5 M KCl solution to 1 ml of medium to achieve an osmotic pressure of 330 mM. For nonionic osmotic regulators such as glucose and sucrose, we added 44.8 μl of a 1.5-M solution to 1 ml of medium to reach 330 mM osmotic pressure.

### Expansion of HSC-NK cells

The medium used for PB-NK cell expansion was similarly employed for the expansion of HSC-NK cells. After 14 days of differentiation and expansion, the NK cell ratio was determined using flow cytometry. Instead of undergoing NK cell purification, the differentiated bulk cells were used directly for HSC-NK cell expansion. These bulk cells, comprising a total of 1 × 10^5^ NK cells, were cocultured with 2 × 10^5^ K562-mbIL21-feeder cells in 1 ml of medium. Twice the volume of the medium was added every three days, taking care not to disrupt the cell aggregates. After 7 days of expansion, the cells were counted and analyzed. For another round of expansion, 1 × 10^5^ cells were cocultured with 2 × 10^5^ K562-mbIL21-feeder cells in 1 ml of medium. Once again, twice the volume of the medium was added every three days, avoiding disruption of the cell aggregates until 7 days of expansion had passed. After a combined total of 14 days of expansion, a functional analysis was conducted.

### Flow cytometry analysis

For flow cytometry, cells were initially washed with PBS before being subjected to staining in PBE buffer (PBS supplemented with 2% FBS and 2 mM EDTA) containing fluorescence-conjugated antibodies. This staining process was carried out at room temperature for 30 min. Following staining, cells were washed twice with PBE buffer and resuspended in PBE for subsequent flow cytometry analysis using a BD FACSCanto II instrument. The data collected were later analyzed using either BD or FlowJo software.

The antibodies used for staining encompassed: CD3 (eBioscience, clone OKT3), CD14 (eBioscience, clone 61D3), CD16 (eBioscience, clone CB16), CD19 (eBioscience, clone HIB19), CD34 (eBioscience, clone 4H11), CD45 (Biolegend, clone HI30), CD56 (eBioscience, clone CMSSB), CD69 (eBioscience, clone FN50), CD94 (eBioscience, clone HP-3D9), CD159a (NKG2A) (Miltenyi Biotec, clone REA110), CD226 (DNAM-1) (eBioscience, clone 11A8.7.4), CD314 (NKG2D) (eBioscience, clone 1D11), CD335 (NKp46) (eBioscience, clone 9 E2), CD336 (NKp44) (eBioscience, clone 44.189), CD337 (NKp30) (eBioscience, clone AF29-4D12), IL21 (eBioscience, clone 3A3-N2), DLL1 (BD, 744833), DLL4 (BD, 564412), HLA-ABC (eBioscience, clone W6/32), along with several isotype control antibodies including Mouse IgG1 κ Isotype Control Antibody (Biolegend, clone MOPC-21), Mouse IgG3 isotype (eBioscience, clone B10), and Rat IgM isotype (eBioscience, clone eBRM).

### Lentivirus transduction in primary cells

The plasmids utilized in this research were assembled using the NEBuilder HiFi DNA Assembly Kit (New England Biolabs) according to previously established methodologies [27]. Production of lentiviral vectors was conducted following a conventional calcium phosphate precipitation protocol. To concentrate the lentiviral vectors, a centrifugation step was performed at 6000 × g for 24 h at 4 °C. This process yielded biological titers ranging between 2 and 10 × 10^7^/ml.

Before the initiation of lentiviral transduction, cord blood HSPCs were cultured for a period of 2 days. The transduction procedure involved the addition of lentivirus at a multiplicity of infection (MOI) of 10 to 1 × 10^5^ cells in 0.5 ml of the culture medium. The medium was supplemented with 8 μg/ml protamine sulfate and 0.1% Poloxamer Synperonic F108. Following transduction, the medium was replaced the next day. The cells were then either immediately used for NK cell differentiation using a standard protocol or maintained in fresh medium until subsequent flow cytometry analysis.

The PB-NK cells were expanded over a period of 6 days using a standard protocol before initiating lentiviral transduction. Here, 1 × 10^5^ cells were transduced with lentivirus at an MOI of 10 in 0.5 ml of culture medium, which was further supplemented with 8 μg/ml protamine sulfate and 0.1% Poloxamer Synperonic F108. After transduction, the medium was replaced with 1 ml of fresh medium, and the cells were cocultured with 2 × 10^5^ K562-mbIL21 feeder cells. Functional analysis of the cells was undertaken after a 7-day expansion cycle or at specified time points during the culture.

### In vitro* tumor cytotoxicity assay*

Post expansion, NK cells (acting as effector cells) and GFP-positive tumor cells (serving as target cells) were cocultured at a designated effector-to-target (E:T) ratio [28]. After 24 h of coculture, the cell population was quantified, and the proportion of GFP-positive tumor cells was assessed via flow cytometry. The degree of cell cytotoxicity was computed using the formula [100 − (GFP cell counts × 100)/Tumor control count]%, where the 'Tumor control count' refers to the number of tumor cells present when not exposed to NK cells.

### RNA-seq analysis

NK cells were enriched utilizing the Human CD56-Positive Selection Kit II (Stemcell Technologies), and the purity of these enriched NK cells was verified via flow cytometry. We extracted total cellular RNA from the enriched NK cells with the aid of the RNeasy Kit (Qiagen).

For RNA sequencing (RNA-seq), we dispatched the samples to Novogene. Sequencing was performed on the Illumina HiSeq 2000 platform, adhering to standard protocols. The DESeq2 package [29] was employed to identify differentially expressed genes (DEGs), with criteria set at a false discovery rate (FDR) less than 0.05 and a fold change (FC) greater than 2. We used the EnhancedVolcano package (version 1.4) to generate volcano plots.

To discern the biological implications of the DEGs, Gene Ontology (GO) analysis was carried out. This analysis was conducted with the help of the goenrich package (1.0.3), which determined significantly enriched GO terms associated with the gene set obtained from the DEG analysis.

### Statistical analysis

The statistical evaluation of the data was executed using either a paired Student's t test or two-way ANOVA, contingent on the data's characteristics (paired/matched or unmatched). *P* value calculations were performed with GraphPad Prism 7.04 software. Adjusted *P* values were specified when applicable. Each figure legend provides a description of the specific statistical methods utilized for their respective experiments. All statistical analyses reported in the study are based on a minimum of three independent experiments. In the context of primary cells, our experiments incorporated samples from at least two distinct donors, bolstering the robustness and generalizability of our findings.

## Results

### *Enhancing NK cell differentiation from cord blood HSPCs *via* osmotic pressure modulation*

**Fig. 1:**
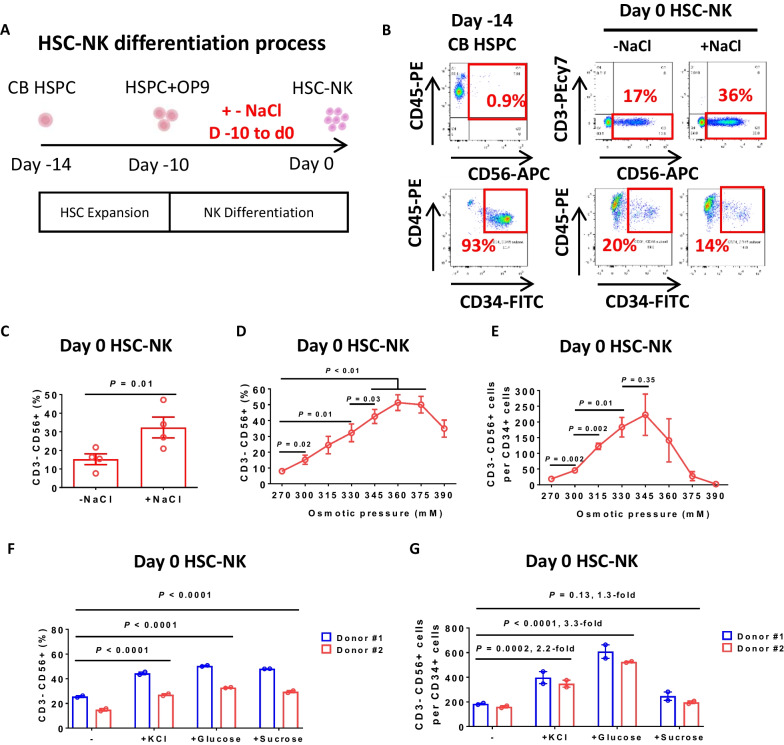
Enhancing NK cell differentiation from hematopoietic stem and progenitor cells through osmotic pressure modulation. **A** A schematic illustration of the HSC-NK cell differentiation protocol beginning with CB HSCs. Following a four-day HSC expansion, HSCs were co-cultivated with OP9-DLL1-DLL4 feeder cells with optional osmotic pressure regulation via the addition of a 9% NaCl water solution (w/v) to the medium. **B** The expression levels of CD34, CD56, and CD3 cells both before and after a 14-day period of HSC expansion and differentiation. **C** Frequency of CD56^+^ CD3^−^ NK cells after 14 days of HSC expansion and differentiation (mean ± SEM, n = 4 independent experimental replicates). High osmotic pressure was manipulated by adding 22.4 μl of a 9% NaCl solution to 1 ml of medium, resulting in a final osmotic pressure of 330 mM. **D** Frequency of CD56^+^ CD3^−^ NK cells after 14 days of HSC expansion and differentiation under the specified osmotic pressure regulation (mean ± SEM, n = 4 independent experimental replicates). **E** The number of CD56^+^ CD3^−^ NK cells generated from a single HSC, as demonstrated in (**D**) (mean ± SEM, n = 4 independent experimental replicates). **F** Frequency of CD56^+^ CD3^−^ NK cells following a 14-day period of HSC expansion and differentiation. The experiments were conducted utilizing HSPCs from two distinct donors, with each experiment replicated technically twice. To achieve a final osmotic pressure of 330 mM, we introduced 22.4 μl of 1.5 M KCl, 44.8 μl of 1.5 M glucose, or 44.8 μl of 1.5 M sucrose into 1 ml of medium. **G** The quantity of CD56^+^ CD3^−^ NK cells generated from a single HSC, as indicated in (**F**), with corresponding fold changes noted. The *P*-values obtained from the paired Student's t-test are indicated in panels (**C**), (**D**), and (**E**), while panels (**F**) and (**G**) underwent a two-way ANOVA analysis

To improve the differentiation of NK cells from HSPCs, we devised a culture system using CD34^+^ HSPCs enriched from cord blood. By employing a combination of cytokines and small molecules, we initiated the differentiation process [21]. We utilized Notch ligand transduced OP9 feeder cells [23] as a supportive environment, promoting the differentiation of HSPCs toward the NK cell lineage (Fig. 1A). By day 14, we observed that approximately 17% of the cells manifested the NK cell phenotype, denoted by CD3^−^ and CD56^+^ expression, while the proportion of CD34-positive cells declined due to differentiation (Fig. 1B, C).

In our 48-well plate-based chemical screening system designed for HSC-NK differentiation, we discerned that the differentiation efficiency in the corner and side wells outpaced that in the center wells. Based on this observation, we conjectured that the increased differentiation efficiency could be attributed to a higher osmotic pressure caused by escalated water evaporation [30]. This led us to hypothesize that increased osmotic pressure could stimulate NK cell differentiation. When we introduced NaCl to the culture medium, thereby inducing 330 mM osmotic pressure, we noted a significant enhancement in NK cell differentiation efficiency (Fig. 1B, C).

Further investigations into the influence of diverse osmotic pressures on differentiation efficiency revealed that the proportion of NK cells escalated with osmotic pressures ranging from 270 to 360 mM before decreasing at 375 mM and 390 mM (Fig. 1D). We also recorded a rise in NK cell output per HSPC from 270 to 330 mM osmotic pressure, which subsequently declined from 345 to 390 mM (Fig. 1E). Other ionic osmotic pressure regulators, such as phosphate-buffered saline (PBS), were found to be similarly effective in controlling NK cell differentiation efficiency (Additional file 1: Fig. S1A–E).

Notably, non-Na^+^ salt solutions, for example, KCl, and nonionic osmotic regulators, such as glucose and sucrose, also significantly elevated the proportion of CD3^−^ CD56^+^ NK cells (Fig. 1F). High osmotic pressure regulated by glucose resulted in a 3.3-fold increase in NK cell output, signifying its potential as a potent inducer of NK cell differentiation (Fig. 1G). Sucrose, while not significantly altering overall NK cell output, led to a noticeable increase in the proportion of NK cells within differentiated cell populations. Taken together, these findings underscore osmotic pressure modulation as a promising and effective approach to enhance NK cell differentiation from HSPCs.

### High osmotic pressure-primed HSC-NK cells show enhanced proliferation activity

**Fig. 2:**
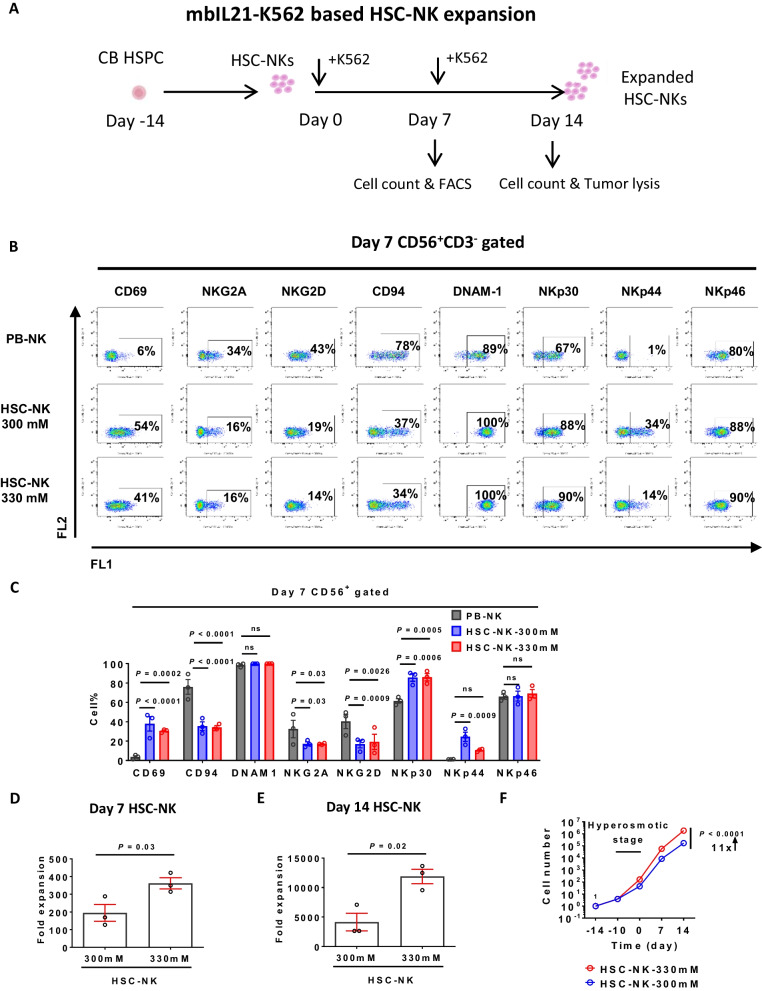
Enhanced proliferation activity in high osmotic pressure-primed HSC-NK cells. **A** A schematic representation of the HSC-NK expansion protocol using K562-mbIL21-feeder cell stimulation. **B** Example data from flow cytometry analysis of surface markers on NK cells after a 7-day NK cell expansion period. **C** Proportions of NK cell marker expression, as illustrated in (**B**). **D** and **E** Fold expansion of CD56^+^ CD3^−^ NK cells after a 7-day (**D**) and 14-day (**E**) expansion period. **F** A growth curve of HSC-NK cells, originated from a single HSC, at the mentioned time points. Hyperosmosis was adjusted by NaCl addition. Data from **C**, **D**, **E**, and **F** are represented as mean ± SEM from 3 independent experimental replicates. *P* values calculated by a paired Student's t-test are provided in (**D**) and (**E**), whereas *P* values computed by a two-way ANOVA test are indicated in (**C**) and (**F**)

As we sought to evaluate the proliferation capability of HSC-NK cells, they were cultured alongside modified K562-mbIL21 feeder cells [22] (Fig. 2A). After the observation that there was no significant variance in the expansion efficiency between FACS-sorted and unsorted HSC-NK cells (data not shown), we streamlined the process by directly culturing differentiated blood cells without isolating CD56^+^ NK cells. For comparison, we adopted peripheral blood-derived NK cells (PB-NK), which are prevalently employed in both preclinical research and clinical therapy [31, 32] (Additional file 1: Fig. S2A). Although HSC-NK cell expansion after 14 days was moderately inferior to that of PB-NK cells (Additional file 1: Fig. S2B), HSC-NK cultures demonstrated superior purity of CD3^−^ CD56^+^ NK cells and fewer CD3^+^ T cells (Additional file 1: Fig. S2C–E).

Our primary examination aimed to determine the effect of osmotic pressure on NK cell purity and phenotype. Both standard osmotic pressure (300 mM) and elevated osmotic pressure (330 mM) conditions facilitated substantial expansion of HSC-NK cells, yielding over 90% purity following a 14-day culture period (Additional file 1: Fig. S2C, E). A noteworthy advantage of HSC-NK cells was their diminished residual presence of CD3-positive cells compared to PB-NK cell cultures (Additional file 1: Fig. S2D, E). We assessed several surface markers linked to NK cells, incorporating both activating and inhibitory receptors [3], such as CD94, NKG2A, CD69, DNAM1, NKG2D, NKp30, NKp44, and NKp46 (Fig. 2B, C). In HSC-NK-300 mM and HSC-NK-330 mM cells, inhibitory receptors such as CD94 and NKG2A were significantly diminished. Conversely, some activating receptors, namely, CD69, NKp30, and NKp44, displayed significant elevations in HSC-NK-300 mM or HSC-NK-330 mM cells. Among the analyzed markers, only NKG2D exhibited a significant decline in both HSC-NK-300 mM and HSC-NK-330 mM cells compared to the PB-NK reference.

Our ensuing investigation was focused on determining the impact of osmotic pressure on NK cell yield. Throughout the culture period, HSC-NK-330 mM cells subjected to hyperosmotic conditions consistently displayed superior proliferation rates compared to their normal osmotic pressure counterparts (HSC-NK-300 mM) (Fig. 2D, E). Considering the entire process from HSPCs to HSC-NK cells, hyperosmosis resulted in an impressive total expansion of 1.8 × 10^6^-fold, which translates to an 11-fold escalation in NK cell output compared to the standard osmotic pressure control (Fig. 2F). Therefore, our observations strongly suggest that hyperosmosis significantly augments the yield of NK cells derived from cord blood sourced HSPCs.

### Transcriptomic profiling reveals activation of HSC-NK cells in response to K562-mbIL21 feeder stimulation

**Fig. 3:**
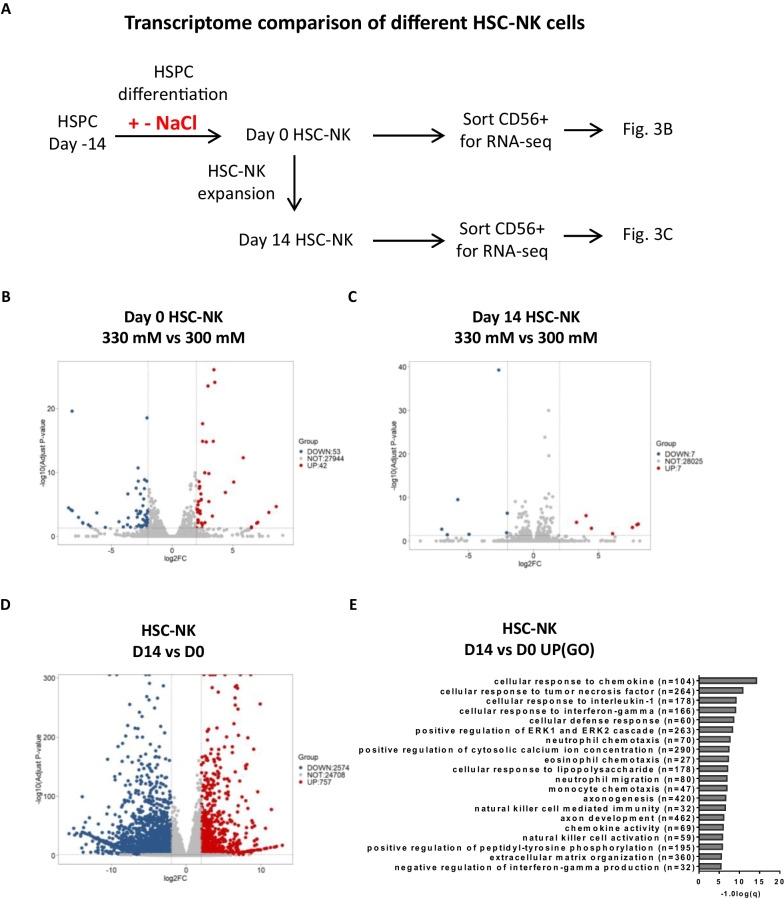
Transcriptomic profiling of HSC-NK cells in response to K562-mbIL21 feeder cell stimulation. **A** Schematic illustrating the RNA sequencing analysis of day 0 and day 14 HSC-NK cells. **B** Volcano plot comparing day 0 HSC-NK cells in a 330 mM osmotic environment to the 300 mM control. **C** Volcano plot comparing day 14 HSC-NK cells in a 330 mM osmotic environment to the 300 mM control. **D** Volcano plot comparing day 14 HSC-NK cells to day 0 HSC-NK cells. The data for each group comprises integrated data from the 300 mM and 330 mM conditions. **E** Gene Ontology (GO) analysis of significantly enriched genes in expanded day 14 HSC-NK cells, as depicted in (**D**). Bar charts illustrate the top 20 significantly enriched GO terms for molecular function, biological process, and cellular components in day 14 HSC-NK cells

To comprehend the traits of HSC-NK cells proliferated under hyperosmotic conditions, we carried out RNA sequencing analysis on HSC-NK-300 mM cells and HSC-NK-330 mM cells (Fig. 3A). The criteria for defining differentially expressed genes were set at an adjusted *P* value (padj) < 0.05. Intriguingly, fewer than 100 differentially expressed genes emerged when HSPCs were subjected to 14 days of differentiation under 330 mM osmotic pressure (Fig. 3B). Analogously, only 14 genes demonstrated differential expression following 14 days of K562-aided expansion of HSC-NK cells (Fig. 3C). These observations imply that high osmotic pressure facilitates more effective differentiation and proliferation of HSC-NK cells without causing considerable alteration to the transcriptome.

Nevertheless, in line with earlier findings, the expanded NK cells displayed distinct gene expression profiles in contrast to their uncultured counterparts, with differential expression seen in more than 3000 genes (Fig. 3D). This indicates an activated transcriptomic profile in the expanded NK cells. The enrichment analysis using Gene Ontology (GO) revealed that the most substantially upregulated signaling pathways in the expanded NK cells were affiliated with natural killer cell activation, immune response, and cytokine responses, which include interferon (IFN)-gamma, interleukin (IL)-1, and tumor necrosis factor (TNF) (Fig. 3E). In sum, these transcriptomic revelations underscore that hyperosmosis does not modify the essential characteristics of highly proliferated HSC-NK cells. Moreover, they suggest the successful activation of HSC-NK cells during K562-modulated expansion, as evidenced by the observed gene expression alterations.

### Hyperosmosis-induced HSC-NK cells exhibit antitumor efficacy comparable to that of PB-NK cells

**Fig. 4:**
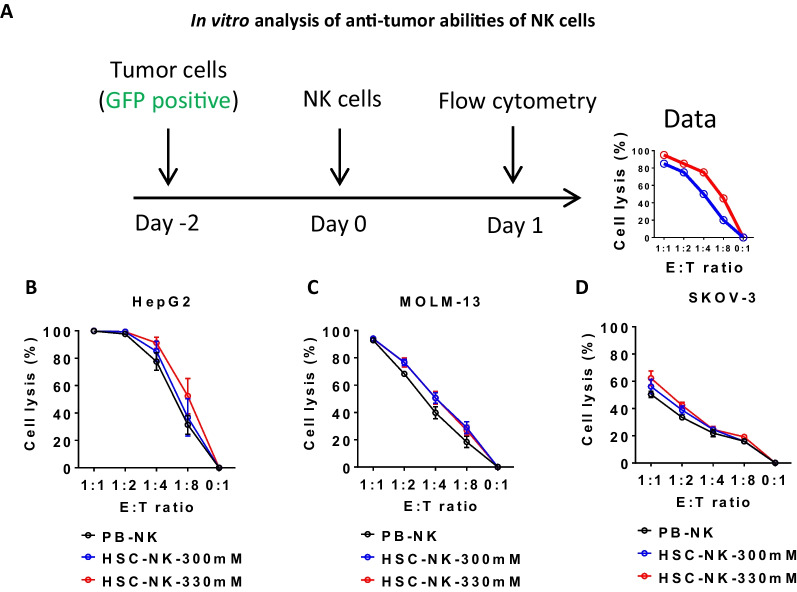
Anti-tumor activity of HSC-NK cells compared to PB-NK cells. **A** Schematic of the in vitro analysis of NK cell anti-tumor abilities. Tumor cells were transduced with a lentiviral vector to express GFP protein. After co-culture of NK cells and tumor cells, the residual tumor cells were analyzed by flow cytometry. **B**–**D** Flow cytometry-evaluated killing activity against HepG2 (B), MOLM-13 (**C**), and SKOV-3 (**D**) after a 24-h co-culture of NK cells and target cancer cells at the indicated E:T ratios. Data in **B**, **C**, and **D** are displayed as mean ± SEM from 3 independent experimental replicates. A two-way ANOVA test was performed, and the results are presented in Additional file 1: Fig. S3

Having established the congruency in the phenotype of NK cells expanded under hyperosmotic pressure, we proceeded to evaluate their functionality. In vitro assessments of the antitumor activity of HSC-NK cells were conducted using HepG2 hepatocellular carcinoma cells, MOLM-13 acute myeloid leukemia cells, and SKOV-3 ovarian cancer cells as targets (Fig. 4A). Predominantly, both HSC-NK-300 mM cells and HSC-NK-330 mM cells exhibited antitumor activity comparable to that of PB-NK cells (Fig. 4B–D). Nevertheless, under specific conditions, such as a 1:8 effector-to-target (E:T) ratio for HepG2 cells, 1:4 and 1:8 E:T ratios for MOLM-13 cells, and 1:1 and 1:2 E:T ratios for SKOV-3 cells, HSC-NK-300 mM and/or HSC-NK-330 mM cells demonstrated noteworthy enhancements in antitumor efficacy compared to PB-NK cells (Additional file 1: Fig. S3). This heightened antitumor activity of HSC-NK cells could be ascribed to their increased expression of certain activating receptors in comparison to PB-NK cells (Fig. 2B, C). Nevertheless, no considerable disparity in cytotoxic activity was discerned between HSC-NK-300 mM and HSC-NK-330 mM cells (Additional file 1: Fig. S3). Thus, these functional observations provide compelling substantiation that HSC-NK cells exhibit antitumor capabilities commensurate with the benchmark established by PB-NK cells.

### Hyperosmosis facilitates the efficient generation of functional HSC-CAR-NK cells

**Fig. 5:**
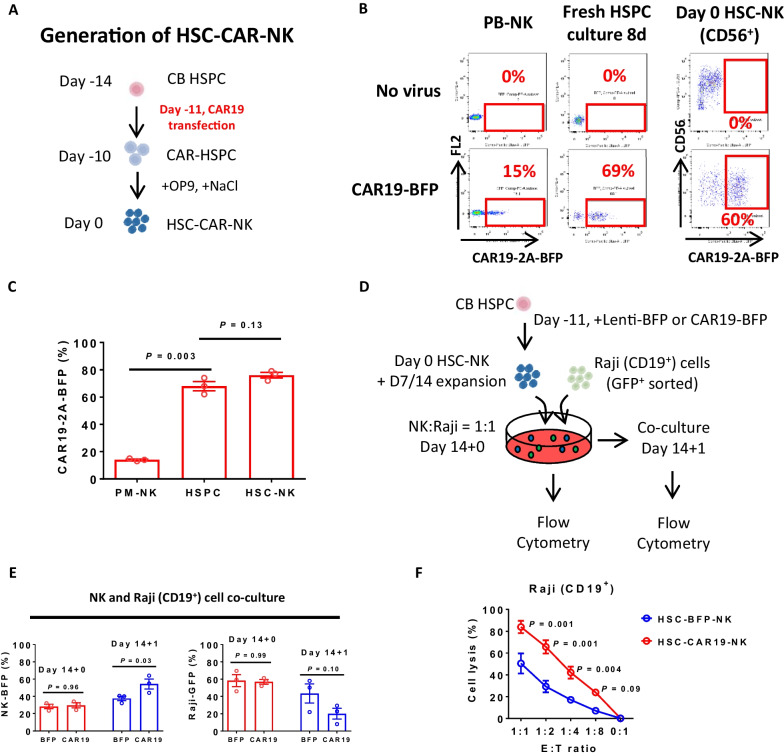
Hyperosmosis facilitates the efficient production of functional HSC-CAR-NK cells. **A** Workflow detailing the production of CAR19 NK cells via HSC differentiation. **B** Flow cytometry assessment of the proportion of BFP-positive cells of PB-NK cells following a 4-day culture and HSPCs following an 8-day culture or NK cell differentiation process, post-transduction with a CAR19-BFP lentiviral vector. The percentages indicate the fraction of BFP-positive cells. **C** Assessment of the fraction of CAR19-BFP positive cells following lentiviral transduction in PB-NK cells, HSPCs as demonstrated in (**B**), and differentiated HSPCs (D0 HSC-NK) bulk populations (bulk cells). **D** Schematic of the co-culture setup of expanded HSC-NK cells with GFP^+^ Raji cells. **E** Proportion of BFP^+^ NK cells and GFP^+^ Raji cells as depicted in (**D**). **F** Flow cytometry-evaluated killing activity against CD19^+^ GFP^+^ Raji cells after a 24-h co-culture of HSC-BFP-NK cells or HSC-CAR19-NK cells and Raji cells at the indicated E:T ratios. Data in **C**, **E** and **F** are represented as mean ± SEM from at least 3 independent experimental replicates. *P* values calculated by a paired Student's t-test are provided in (**C**), whereas *P* values computed by a two-way ANOVA test are indicated in (**E**) and (**F**)

NK cells, which have been genetically engineered to express a CAR, show promising clinical responses in cancer patients when utilized in adoptive cellular immunotherapy. We centered our attention on an anti-CD19 CAR (CAR19), comprising an anti-CD19 extracellular single-chain variable fragment and a CD3z signaling domain. We successfully developed an efficacious method for generating functional CAR19-NK cells through hyperosmosis from HSPC differentiation (Fig. 5A).

To enable the detection of transduced CAR-NK cells, we designed a lentivirus expressing a CAR19-2A-BFP construct. Consequently, CAR19-expressing cells were also positive for blue fluorescent protein (BFP), thereby enabling their identification through flow cytometry. As a control, we utilized BFP expression without CAR19. During the HSPC expansion phase, the lentivirus successfully transduced HSPCs (Fig. 5B and Additional file 1: Fig. S4B), displaying notably higher efficiency than peripheral blood-derived NK (PB-NK) cells (Fig. 5C and Additional file 1: Fig. S4A). Upon differentiation into HSC-CAR-NK cells, the proportion of CAR19-transduced cells remained consistent with that of undifferentiated HSPCs (Fig. 5B, C).

Remarkably, hyperosmosis significantly amplified both the percentage and total count of CD3^−^ CD56^+^ cells after 14 days of HSPC expansion and differentiation, irrespective of lentivirus transduction (Additional file 1: Fig. S4C, D). Additionally, these findings lend support to the assertion that lentivirus-mediated CAR transduction and differentiation of HSC-CAR-NK cells do not appreciably impact the output cell numbers of NK cells (Additional file 1: Fig. S4D). This advantage of HSC-CAR-NK cells is particularly valuable because it potentially circumvents the challenges tied to relatively lower cell viabilities observed in PB-NK cells after lentivirus transduction, especially at a high multiplicity of infection (MOI). Hence, HSC-CAR-NK cells hold promise for surmounting these limitations and ensuring robust cell expansion and viability for successful CAR-NK therapy applications.

CAR19-NK cells are designed to amplify their cytotoxic activity against CD19^+^ tumor cells. In our study, we explored the targeting capability of HSC-CAR-NK cells against Raji cells, which are CD19 positive and exhibit high levels of HLA class I molecules. Conversely, K562 cells are CD19 negative and display low levels of HLA class I molecules (Additional file 1: Fig.  S4E). Raji cells, owing to their elevated expression of HLA class I molecules, demonstrate resistance to NK cells lacking CAR19 modification.

To evaluate the targeting activity of HSC-CAR-NK cells against Raji cells, we expanded HSC-NK cells transduced with either BFP or CAR-2A-BFP and initiated coculture with GFP-positive Raji cells (Fig. 5D). Upon quantification of the percentages of BFP-positive and GFP-positive cells, we observed a significant surge in BFP-positive cells in HSC-CAR-NK cells relative to the control HSC-BFP-NK cells on the first day of coculture with CD19^+^ Raji cells (Fig. 5E and Additional file 1: Fig. S4F). Additionally, under 1:1, 1:2, and 1:4 E:T ratios conditions, HSC-CAR19-NK cells demonstrated significant enhancement in cell lysis efficacy of CD19^+^ Raji cells compared to HSC-BFP-NK cells (Fig. 5F). In summary, these results suggest that CAR19-expressing HSC-NK cells exhibit specific targeting activity against CD19-positive cells.

## Discussion

This study demonstrated how a simple optimization strategy involving an increase in culture medium osmotic pressure significantly enhanced the differentiation efficiency and yield of NK cells derived from human cord blood stem cells. Importantly, these hyperosmosis-induced HSC-NK cells retained their phenotype and maintained their ability to kill tumor cells.

Environmental stress, such as hyperosmosis, can influence cell fate through intracellular signaling and epigenetic modulation [33]. Although the precise mechanisms driving hematopoietic cell differentiation remain elusive, it is hypothesized that a global epigenetic shift underpins this process. Our strategy of enhancing osmotic pressure in the culture medium using NaCl or PBS seems to facilitate NK cell differentiation, thereby enriching the standard NK cell differentiation protocol [34]. While the intricate details of this mechanism are yet to be unveiled, we anticipate that our optimized method will expedite the development of an 'off-the-shelf' source for clinical use [35].

Drawing from our accumulated expertise, we conducted our experiments within the central eight wells of a 24-well tissue culture (TC) plate. We filled the corner and side wells with water to curtail evaporation in the central wells. Recognizing the importance of controlling baseline evaporation when using diverse culture wells or bioreactors is paramount. While the intricate mechanisms involved are yet to be fully explicated, we posit the involvement of stress-related signaling pathways, notably the p38 pathway. This pathway has been reported to respond to augmented osmotic pressure during somatic cell reprogramming [30]. The critical role of cytokine profiles in influencing NK cell differentiation has been previously reported [36]. Alterations in these profiles could reciprocally impact NK cell differentiation, a notion that is in alignment with our own findings, thereby strengthening the idea that environmental modifications, such as encapsulation or osmotic pressure, can markedly affect cell fate decisions. This raises an intriguing question as to whether a synergistic combination of these strategies, such as encapsulation and hyperosmotic pressure, could potentially enhance NK cell differentiation and yield.

The use of allogenic sources such as cord blood for NK cells has shown its safety in the context of immunotherapy [12]. However, the clinical translation potential of these sources is significantly restricted due to the limited number of NK or CAR-NK cell doses they can produce. Specifically, NK cells from a single CB unit or PB donor can typically produce approximately 100 doses. In stark contrast, our optimized protocol allows generation of over 2000 doses of HSC-NK cells within a 28-day period, presuming an administration of 1 × 10^7^/kg for a 50-kg patient.

NK cells, serving as innate immune defenders against viral infections, exhibit resistance to viral vector transduction, even when subjected to an exceptionally high multiplicity of infection (MOI). In a clinical trial involving CB-derived NK cells engineered to express a CAR cassette, the final transduction efficiency was observed to be 49%, varying from 22.7 to 66.5% [12]. When we employed peripheral blood-derived NK (PB-NK) cells, the lentiviral transduction efficiency was between 15 and 20%.

In contrast, hematopoietic stem cells are considerably more amenable to transduction by lentiviruses or retroviruses [37, 38]. This facet has significant implications for the HSC-NK cell platform. Following viral transduction, hematopoietic stem cells (HSCs) can proliferate and differentiate into hundreds of NK cells, considerably reducing the quantity of virus required for a clinical trial. This crucial efficiency would substantially streamline the product development process, decrease cell therapy costs, and expedite the clinical adoption of NK therapies.

These collective benefits position HSC-CAR-NK cells as a compelling alternative for immunotherapy, directly competing with the currently popular iPSC-CAR-NK strategy. Given their higher transduction efficiency and cost-effectiveness, the use of HSC-CAR-NK cells in immunotherapy appears to be a highly promising approach.

Admittedly, our current protocol has limitations. For instance, we relied on mouse OP9 stromal cells to support HSPC proliferation and NK cell differentiation [23]. Future studies should explore other optimization avenues, such as feeder-free or humanized feeder cell-dependent 3D differentiation systems. Furthermore, while hyperosmosis significantly improved NK cell yield on a small scale, its effectiveness on a larger scale in bioreactors for clinical development warrants further investigation.

An 'off-the-shelf' source can also be differentiated from human iPSCs [13, 14, 34, 35]. This strategy allows precise genetic modifications at the clonal level in iPSCs using both viral [13, 14] and nonviral [34] methods. We conjecture that high osmotic pressure could enhance the differentiation efficiency from pluripotent stem cells to NK cells. Additionally, the potential of hyperosmosis to improve targeted differentiation of iPSCs to HSPCs is worth exploring.

## Conclusions

Our study reveals that high osmotic pressure dramatically improves the differentiation and proliferation of NK cells from hematopoietic stem cells such as cord blood HSPCs. This opens avenues for the mass production of NK cells for clinical use. Combining this technique with innovations such as CAR for tumor targeting and using 'off-the-shelf' iPSCs or HSPCs, we aim to develop potent NK cell therapies for next-generation immunotherapy. Our findings underscore the potential of hyperosmotic regulation in crafting effective, high-yield NK cell treatments for cancers.

### Supplementary Information

**Additional file 1** contains data related to the main text and figures.

## Data Availability

All data supporting the findings of this research are openly accessible in the SRA database under accession number PRJNA983559. The authors confirm the availability of supporting data within this article and its supplemental information.
