# Supplementary material for: Enhancing cord blood stem cell-derived NK cell growth and differentiation through hyperosmosis
Source: Stem Cell Res Ther. 2023 Oct 15;14:295. doi: 10.1186/s13287-023-03461-x (PMC10578005; doi:10.1186/s13287-023-03461-x)
Supplement: Supplementary file 1 — Additional file 1 contains data related to the main text and figures. [file 13287_2023_3461_MOESM1_ESM.pdf]

Supplementary Figure 1

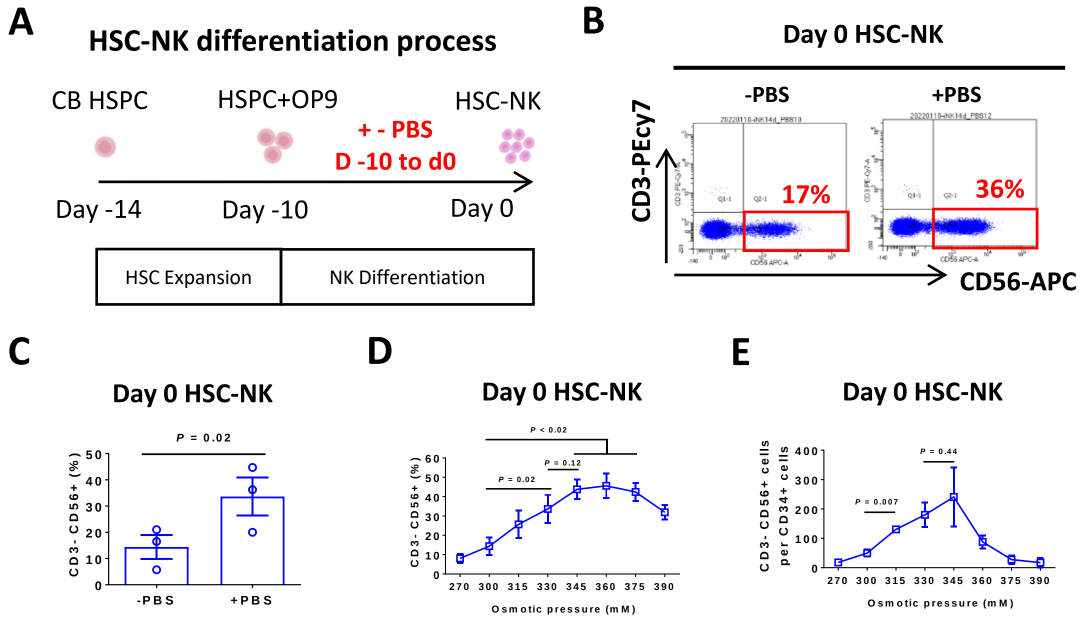

Supplementary Figure 1. Enhanced NK cell differentiation from hematopoietic stem and progenitor cells through osmotic pressure modulation, related to Figure 1. (A) A schematic diagram of the HSC-NK differentiation protocol, which starts with cord blood (CB) HSCs. After a 4-day HSC expansion, the HSCs were cocultured with OP9-DLL1-DLL4 feeder cells, with the option of osmotic pressure regulation by adding a 10x PBS solution to the medium. (B) Expression levels of CD56 and CD3 cells following a 14-day period of HSC expansion and differentiation. (C) Frequency of CD56+ CD3- NK cells after 14 days of HSC expansion and differentiation (mean ± SEM, n = 3 independent experimental replicates). High osmotic pressure was achieved by adding 22.4 µl of 10x PBS solution to 1 ml of medium, resulting in a final osmotic pressure of 330 mM. (D) Frequency of CD56+CD3- NK cells after 14 days of HSC expansion and differentiation under the indicated osmotic pressure regulation (mean ± SEM, n = 3 independent experimental replicates). (E) Numbers of CD56+ CD3- NK cells generated from a single HSC, as indicated in (D) (mean ± SEM, n = 3 independent experimental replicates). *P* values were analyzed by paired Student‘s t-test in (C), (D), and (E).

Supplementary Figure 2

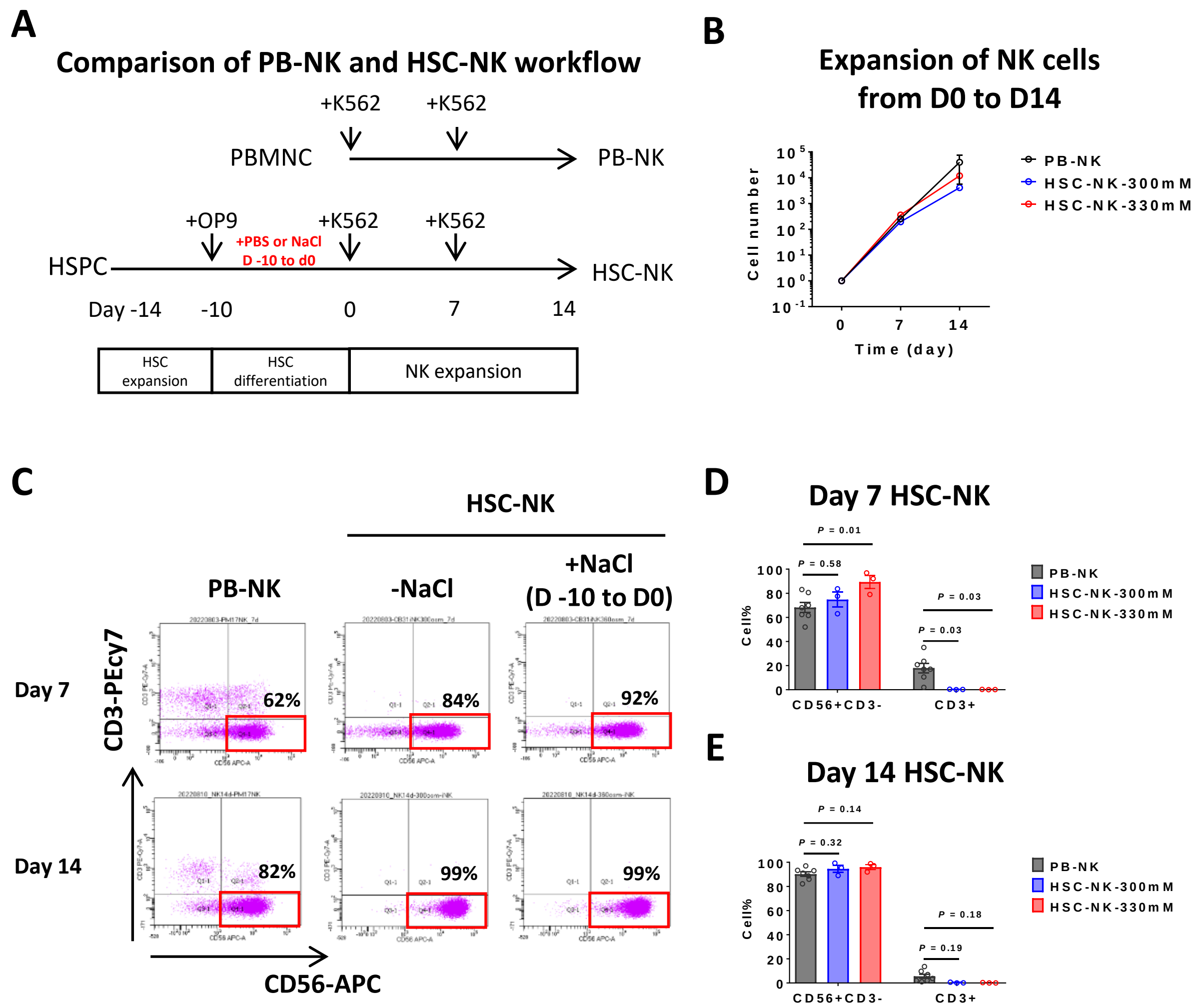

Supplementary Figure 2. Enhanced proliferation activity of high osmotic pressure-primed HSC-NK cells, related to Figure 2. (A) Workflow comparison between PB-NK cells and differentiated HSC-NK cells. (B) Growth curves of PB-NK cells and differentiated HSC-NK cells following K562-mbIL21-based expansion. (C) Expression levels of CD3 and CD56 after periods of 7 and 14 days. (D and E) Proportion of CD56+CD3- cells and CD3+ cells after 7 days (D) and 14 days (E) of expansion, as presented in (C). Data in (B), (D), and (E) are represented as mean ± SEM from at least 3 independent experimental replicates. *P* values calculated by a two-way ANOVA test are indicated in (D) and (E).

Supplementary Figure 3

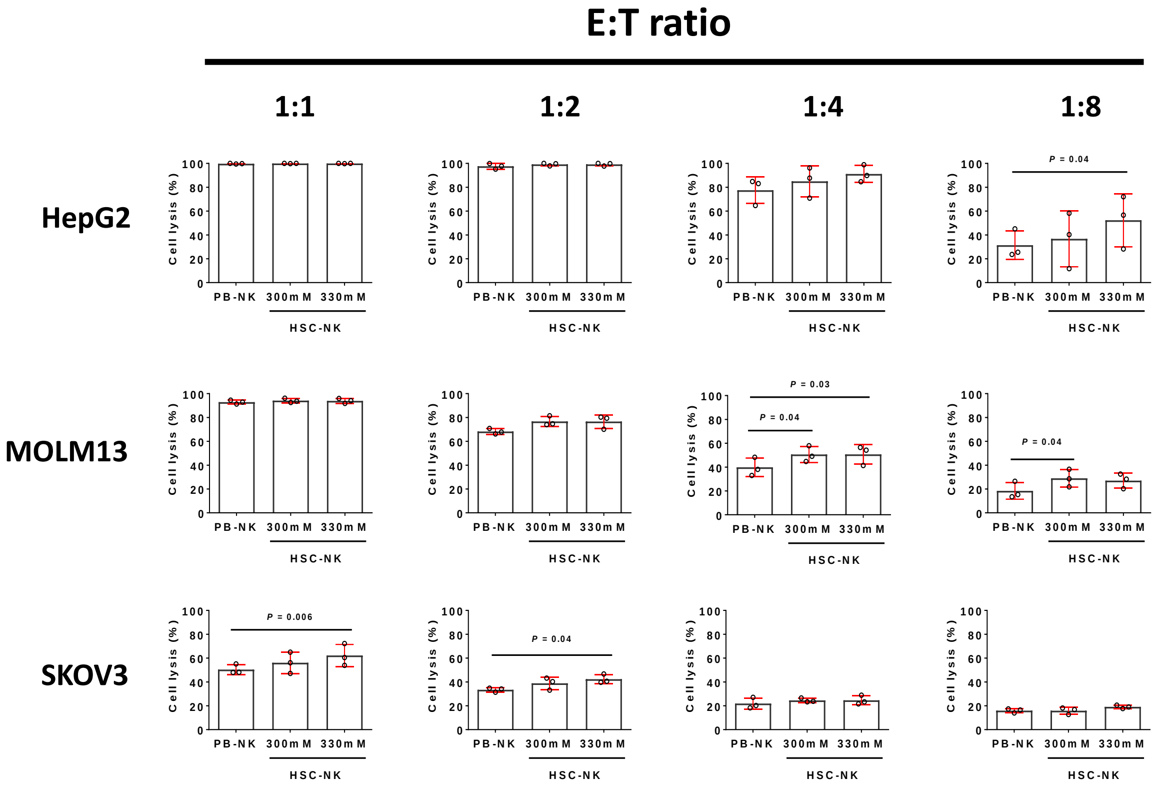

Supplementary Figure 3. Anti-tumor activity of HSC-NK cells versus PB-NK cells, related to Figure 4. The graph shows individual kill efficiencies of different NK cells under indicated conditions. HSC-NK cells, expanded under both normal and hyper osmotic conditions, demonstrated equal or superior killing activity to PB-NK control cells against three distinct cancer cell lines at E:T ratios ranging from 1:1 to 1:8. The data is formatted similarly to Figure 4, and significant *P* values ($P < 0.05$) mentioned in Figure 4 are indicated.

Supplementary Figure 4

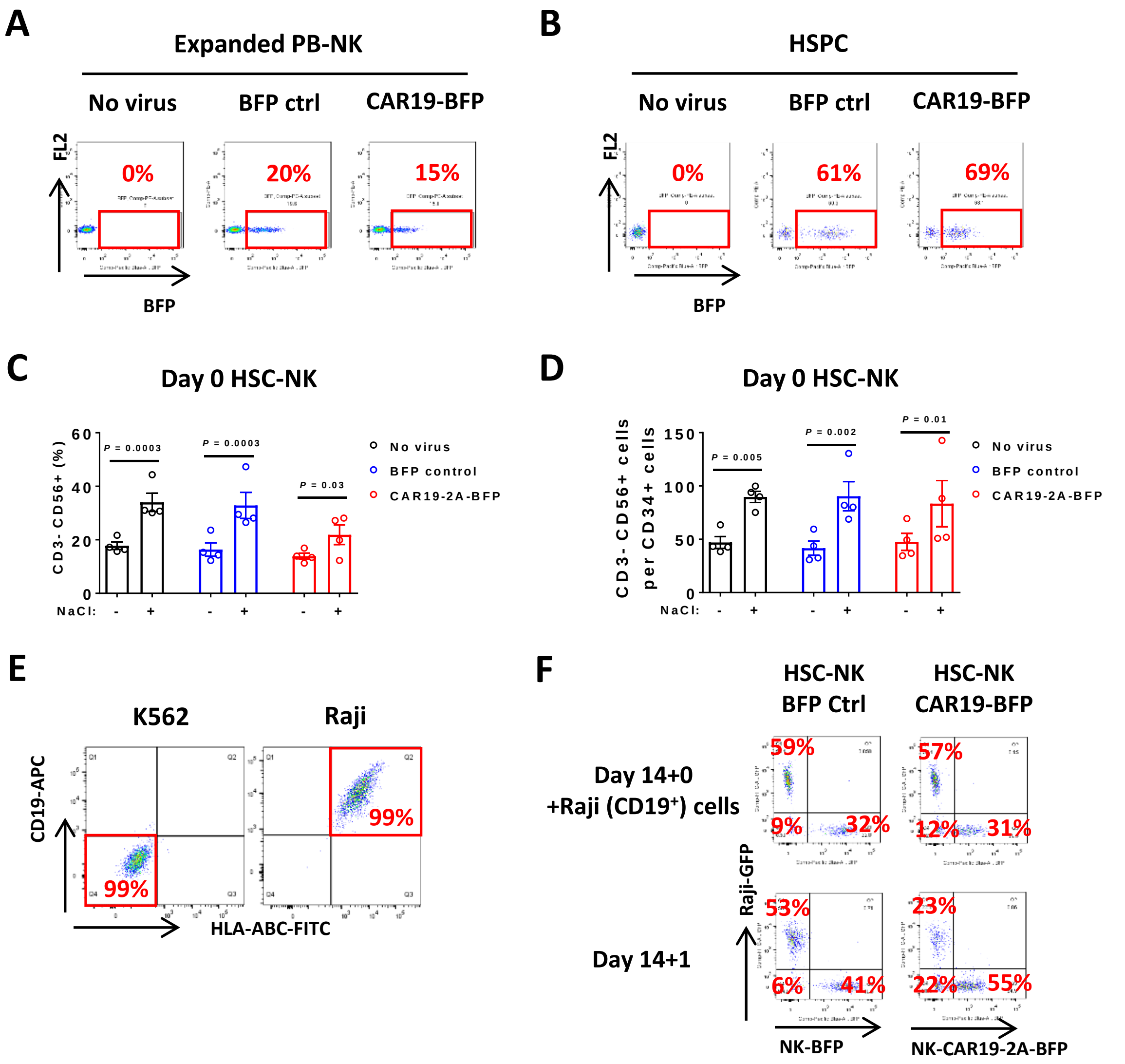

Supplementary Figure 4. Hyperosmosis augments the production of functional HSC-CAR-NK cells, related to Figure 5. (A and B) Efficacy of lentiviral transduction expressing BFP or CAR19-BFP in PB-NK cells (A) and HSPCs (B). (C) Proportion of CD56+CD3- NK cells following a 14-day period of HSPC expansion and differentiation, with or without lentiviral transduction. High osmotic pressure was achieved by the addition of 22.4 µl of a 9% NaCl solution to 1 ml of medium, leading to a final osmotic pressure of 330 mM. (D) Enumeration of CD56+CD3- NK cells originating from a single HSPC, as shown in (C), expressed as mean ± SEM from 4 independent experimental replicates.). (E) Flow cytometry analysis showing the expression of CD19 and HLA class I molecules (HLA-ABC) in K562 and Raji cells. The percentages in red denote the double negative and double positive expression of CD19 and HLA-ABC in K562 and Raji cells, respectively. (F) Flow cytometry analysis of BFP+ NK cells and GFP+ Raji cells at the initiation (day 14+0) or after 24 hours (day 14+1) of co-culture as illustrated in figure 5D. Statistical significance was determined via a two-way ANOVA in (C) and (D).
